# Treatment of Cancer-Associated Thrombosis: Recent Advances, Unmet Needs, and Future Direction

**DOI:** 10.1093/oncolo/oyad116

**Published:** 2023-05-12

**Authors:** Tzu-Fei Wang, Alok A Khorana, Giancarlo Agnelli, Dan Bloomfield, Marc P Bonaca, Harry R Büller, Jean M Connors, Shinya Goto, Zhi-Cheng Jing, Ajay K Kakkar, Yasser Khder, Gary E Raskob, Gerald A Soff, Peter Verhamme, Jeffrey I Weitz, Marc Carrier

**Affiliations:** Department of Medicine, University of Ottawa, The Ottawa Hospital, Ottawa Hospital Research Institute, Ottawa, Canada; Department of Hematology and Medical Oncology Taussig Cancer Institute Cleveland Clinic Foundation, Cleveland, OH, USA; Internal Vascular and Emergency Medicine - Stroke Unit, University of Perugia, Perugia, Italy; Anthos Therapeutics, Cambridge, MA, USA; Department of Medicine, Division of Cardiology, University of Colorado School of Medicine, Aurora, CO, USA; CPC Clinical Research, Aurora, CO, USA; Department of Vascular Medicine, Academic Medical Center, University of Amsterdam, Amsterdam, The Netherlands; Hematology Division, Brigham and Women’s Hospital, Boston, MA, USA; Department of Medicine (Cardiology), Tokai University School of Medicine, Isehara, Japan; Department of Cardiology, State Key Laboratory of Complex Severe and Rare Diseases, Peking Union Medical College Hospital, Chinese Academy of Medical Sciences and Peking Union Medical College, Beijing, China; Thrombosis Research Institute, London, UK; Anthos Therapeutics, Cambridge, MA, USA; Hudson College of Public Health University of Oklahoma Health Sciences Center Oklahoma City, OK, USA; General Hematology Service, University of Miami Health System/Sylvester Comprehensive Cancer Center, Miami, FL, USA; Department of Cardiovascular Sciences, Vascular Medicine and Hemostasis, KU Leuven, Leuven, Belgium; Thrombosis and Atherosclerosis Research Institute, McMaster University, Hamilton, Canada; Department of Medicine, University of Ottawa, The Ottawa Hospital, Ottawa Hospital Research Institute, Ottawa, Canada

**Keywords:** neoplasms, venous thromboembolism, anticoagulants, factor XIa inhibitors, heparin, low-molecular-weight heparin, factor XI

## Abstract

Cancer-associated thrombosis, with the incidence rising over the years, is associated with significant morbidity and mortality in patients with cancer. Recent advances in the treatment of cancer-associated venous thromboembolism (VTE) include the introduction of direct oral anticoagulants (DOACs), which provide a more convenient and effective option than low-molecular-weight heparin (LMWH). Nonetheless, important unmet needs remain including an increased risk of bleeding in certain patient subgroups such as those with gastroesophageal cancer, concerns about drug-drug interactions, and management of patients with severe renal impairment. Although DOACs are more convenient than LMWH, persistence can decline over time. Factor XI inhibitors have potential safety advantages over DOACs because factor XI appears to be essential for thrombosis but not hemostasis. In phase II trials, some factor XI inhibitors were superior to enoxaparin for the prevention of VTE after knee replacement surgery without increasing the risk of bleeding. Ongoing trials are assessing the efficacy and safety of factor XI inhibitors for the treatment of cancer-associated VTE.

## Teaser

This narrative review summarizes advances in the treatment of cancer-associated venous thromboembolism, outlines key unmet needs with the current treatment, and discusses how factor XI inhibitors may address the current knowledge gaps.

**Implications for Practice**

The incidence of cancer-associated thrombosis has been rising over the years, and this complication is associated with significant morbidity and mortality in patients with cancer. Recent advances in the treatment of cancer-associated thrombosis include the introduction of direct oral anticoagulants (DOACs), which provide a more convenient and effective option than low-molecular weight heparin. Nonetheless, important unmet needs remain, including an increased risk of bleeding in certain patient subgroups, concerns about drug-drug interactions, management of patients with severe renal impairment, and adherence. The newer generation of anticoagulants inhibiting factor XI (FXI) have potential safety advantages over DOACs because factor XI appears to be essential for thrombosis but is mostly dispensable for hemostasis. Phase III trials assessing the efficacy and safety of FXI inhibitors for the management of cancer-associated thrombosis have been initiated.

## Introduction

Patients with cancer are at high risk of venous thromboembolism (VTE), which mainly includes upper or lower extremity deep vein thrombosis and pulmonary embolism (PE).^1^ The 6-month VTE risk in patients with cancer is up to 12-fold higher than that in the general population and up to 23-fold higher in patients with cancer receiving chemotherapy or targeted therapy.^2^ The risk of VTE depends on the type of cancer and is highest in patients with pancreatic cancer, lymphoma (Hodgkin and non-Hodgkin) and ovarian cancer, and lower in those with melanoma.^2^ Patient and treatment-related characteristics also affect the risk of VTE. Prior VTE, metastatic disease, and the use of chemotherapy or targeted therapy increase the risk.^2,3^ Additionally, biomarkers such as d-dimer, soluble p-selectin, and more are independent predictive risk factors for cancer-associated VTE.^3^ Finally, the 12-month cumulative incidence of VTE over the last 2 decades has increased 3-fold among patients with cancer and up to 6-fold in those receiving chemotherapy or targeted therapy.^2^ Therefore, VTE is an increasingly common problem in the cancer population.

VTE in patients with cancer is associated with significant morbidity, hospitalizations, and can interfere or delay potentially curative cancer treatments such as surgery.^4,5^ VTE and arterial thromboembolism have been reported as the second most common cause of death in cancer patients after tumor progression.^4,6^ Furthermore, patients with cancer are more likely to have bleeding complications (12-month cumulative incidence of major bleeding: 12.4%, 95% confidence interval [CI] 6.5-18.2) and recurrent VTE despite anticoagulation therapy (incidence rate: 9.6 [95% CI 8.8-10.4] per 100 ­person-years) compared with those without cancer.^7,8^ Both recurrent VTE and bleeding complications are associated with lower quality of life and an increase in healthcare costs.^5,7^ The diagnosis of VTE can be distressing for patients, particularly in those with little support or knowledge about its diagnosis and treatment.^9^ It increases awareness of worsened prognosis, impacts the family dynamics, and can be an important financial stressor for these vulnerable patients already burdened and often overwhelmed by the substantial costs incurred by their cancer treatment.^9-12^ The purpose of this narrative review is to summarize the advances in the treatment of ­cancer-associated VTE, outline key unmet needs with the current treatment, and discuss how factor XI inhibitors may address the current knowledge gaps.

## Evolution of the Treatment of  Cancer-Associated VTE

Historically, treatment of acute VTE started with a rapidly acting parental anticoagulant (low-molecular-weight heparin [LMWH], fondaparinux, or unfractionated heparin) overlapped with and followed by a vitamin K antagonist (VKA) such as warfarin. However, in patients with cancer, this approach is problematic because of multiple drug-drug interactions (DDIs) with VKAs that reduce or potentiate their anticoagulant effects and unpredictable gastrointestinal (GI) absorption because of vomiting, diarrhea, or malnutrition. The long half-life of VKAs also complicates treatment in the setting of thrombocytopenia or procedures which are common in patients with cancer. The consortium linking oncology to thrombosis trial was the first randomized controlled trial to demonstrate that LMWH (dalteparin) monotherapy was more effective than warfarin for the prevention of recurrence in patients with cancer-associated VTE and did not significantly increase the risk of bleeding.^13^ A meta-analysis of 11 trials comparing LMWH with VKAs for treatment of ­cancer-associated VTE (including consortium linking oncology to thrombosis, CATCH, and others, *N* = 2777) confirmed the superiority of LMWH over VKAs in preventing recurrent VTE (LMWH vs. VKAs, risk ratio [RR] 0.58, 95% CI 0.45-0.75), with a comparable risk of major bleeding (RR 0.99, 95% CI 0.67-1.45).^14,15^ Hence, LMWH became the standard of care for treatment of cancer-associated  VTE until the introduction of the direct oral anticoagulants (DOACs). However, LMWH is expensive, requires burdensome once or twice daily subcutaneous injections, and is associated with declining persistence over time.^16^ These limitations could explain why, until the introduction of the DOACs, VKAs continued to be used in patients with  cancer-associated thrombosis.^16^

Over the past decade, the DOACs have become the anticoagulants of choice for treatment and prevention of VTE in the general population because unlike VKAs, they can be given in fixed doses without routine anticoagulation monitoring.^17^ A meta-analysis of the pivotal phase III trials that compared the DOACs with VKAs in patients with VTE (*N* = 27 023) revealed a similar rate of recurrence but a 40% lower rate of major bleeding with the DOACs than VKAs.^18^ These trials included 1582 patients (5.9%) with active cancer. Analysis in the cancer subgroup showed that compared with VKAs, the DOACs were associated with a significant reduction in recurrent VTE (RR 0.57, 95% CI 0.36-0.91) with no significant difference in major bleeding (RR 0.77, 95% CI 0.44-1.33).^18^ However, the definition of cancer status varied among different studies, and the patients with cancer included in these studies were likely at lower risk than those enrolled in trials comparing VKAs with LMWH because the rates of recurrent VTE and major bleeding risk in these patients with cancer were lower.^19^ Nonetheless, these promising results prompted trials comparing DOACs with LMWH in patients with ­cancer-associated VTE.

Since 2018, 5 such RCTs compared DOACs with dalteparin for treatment of cancer-associated VTE (Table 1).^20-24^ The Hokusai VTE Cancer trial evaluated edoxaban, Select-D and CASTA DIVA evaluated rivaroxaban, and ADAM VTE and CARRAVAGIO evaluated apixaban.^20-24^ Metaanalyses of these RTCs showed that DOACs significantly reduced the risk of recurrent VTE compared with dalteparin (hazard ratio [HR] 0.63, 95% CI, 0.47-0.86), but were associated with an increased, yet not statistically significant, risk of major bleeding (HR 1.26, 95% CI, 0.84-1.90) and a significantly increased risk of clinically relevant non-major bleeding (HR 1.48, 95% CI, 1.18- 1.85).^24,25^ Major international guidelines have since then incorporated DOACs as one of the anticoagulants of choice in most patients with cancer except for those considered at high risk of bleeding with a particular emphasis on unresected GI and potentially genitourinary (GU) malignancies.^26,27^

**Table 1.** Randomized controlled trials of DOACs versus LMWH.

Trials	Hokusai VTE cancer^20^	Select-D^21^	ADAM VTE^22^	Caravaggio^23^	CASTA DIVA^24^
DOACs	Edoxaban	Rivaroxaban	Apixaban	Apixaban	Rivaroxaban
LMWH	Dalteparin	Dalteparin	Dalteparin	Dalteparin	Dalteparin
*N*	1046	406	287	1155	158
Follow-up	12 months	6 months	6 months	6 months	3 months
VTE (%)^*^	6.5 vs. 8.8	3.9 vs. 8.8	0.7 vs. 6.3	5.6 vs. 7.9	6.4 vs. 10.1
	HR 0.75 (0.48-1.17)	HR 0.43 (0.19-0.99)	HR 0.099 (0.01-0.78)	HR 0.63 (0.37-1.07)	SHR 0.75 (0.21-2.66)
MB (%)^*^	5.6 vs. 3.2	5.4 vs. 3.0	0 vs. 1.4	3.8 vs. 4.0	1.4 vs. 3.7
	HR 1.74 (0.95-3.18)	HR 1.83 (0.68-4.96)	HR not estimable	HR 0.82 (0.40-1.69)	SHR 0.36 (0.04-3.43)
CRNMB (%)^*^	12.3 vs. 8.2	12.3 vs. 3.5	6.2 vs. 4.9	9.0 vs. 6.0	10.8 vs. 6.1
	HR 1.55 (1.05-2.28)	HR 3.76 (1.63-8.69)	NR	HR 1.42 (0.88-2.30)	NR
Mortality (%)^*^	26.8 vs. 24.2	23.6 vs. 27.6	16 vs. 11	23.4 vs. 26.4	25.7 vs. 23.8
	HR 1.14 (0.90-1.45)	NR	HR 1.40 (0.82-2.43)	HR 0.82 (0.62-1.09)	HR 1.05 (0.56-1.97)

^*^Rates are DOAC versus LMWH, at 6 months except for CASTA DIVA where follow-up was 3 months; HR is followed by 95% confidence interval in the parenthesis.

Abbreviations: CRNMB, clinically relevant non-major bleeding; DOAC, direct oral anticoagulant; HR, hazard ratio; LMWH, low-molecular weight heparin; MB, major bleeding; NR, not reported; SHR, subdistribution hazard ratio; VTE, venous thromboembolism.

## Knowledge Gaps and Unmet Needs in the Current Management of Cancer-Associated VTE

**Figure 1.**
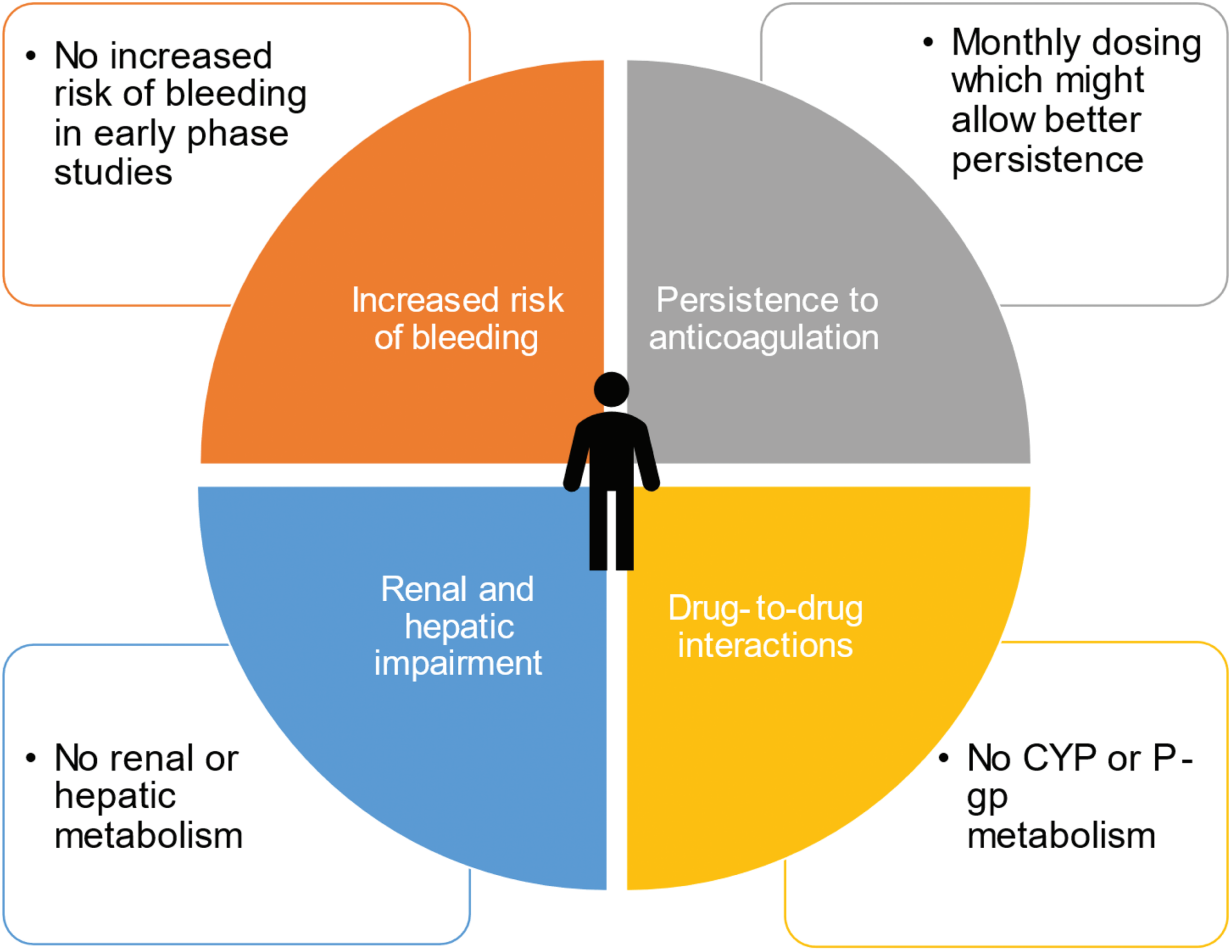
Knowledge gaps and unmet needs related to current anticoagulants for the management of cancer-associated thrombosis (center) and how factor XI inhibitors could address these issues (peripheral).

DOACs are not without problems (Fig. 1). In the Hokusai VTE cancer and Select-D trials, the risk of major hemorrhage was greater with edoxaban or rivaroxaban than with dalteparin.^20,21,28^ Although the rate of major bleeding with apixaban was similar to that with dalteparin in the 2 more recent trials,^22,23^ a meta-analysis demonstrated a 4.3% risk of major bleeding with DOACs at 6 months and an increased risk of major GI bleeding with DOACs compared with LMWH, especially in patients with unresected luminal GI tumors.^25^ There was also an increased risk of major GU bleeding associated with DOACs compared with LMWH.^29^ Recent guidelines have embraced these findings and suggest LMWH over DOACs in these patients, a vulnerable population at particularly high risk of bleeding with unmet needs for a safer anticoagulant option.^26,27^

There are other considerations with the current anticoagulation management for patients with cancer-associated VTE, including potential DDIs with DOACs, especially with medications affecting the cytochrome (CYP) 3A4 and P-glycoprotein (P-gp) pathways, which can increase the risks of bleeding or thrombotic complications.^30-32^ Many drugs used for cancer treatment affect these pathways and may be a concern in patients taking DOACs, as summarized extensively in previous reviews.^30,31,33^ However, much of the evidence remains to be based on pharmacokinetic and pharmacodynamic data only, and more clinically relevant data are needed.^34^ In addition, renal or hepatic dysfunction are common in cancer patients. Renal dysfunction can lead to drug accumulation, which can increase the risk of bleeding with DOACs. Severe liver dysfunction is also problematic because the DOACs are metabolized in the liver and their efficacy and safety in patients with severe liver disease have not been evaluated.

Another problem with the DOACs is the lack of data on the efficacy and safety of the lower dose regimens of rivaroxaban or apixaban for secondary prevention in patients with active cancer or in those at high risk for recurrence. The ongoing trial comparing the treatment and prophylactic doses of apixaban for secondary prevention in patients who have received at least 6 months of full-dose treatment for cancer-associated VTE is designed to address this question (API-CAT trial, NCT03692065).^35^ Finally, because patients with ­cancer-associated VTE often require prolonged anticoagulation therapy, persistence to the DOACs taken once or twice daily often decreases over time, which can lead to VTE recurrence.^16^ Therefore, anticoagulants that are safer than the DOACs, have no DDIs, are not metabolized in the liver or cleared via the kidneys, and can be given once monthly would simplify the treatment of cancer-associated VTE.

## Next-Generation Anticoagulants

**Figure 2.**
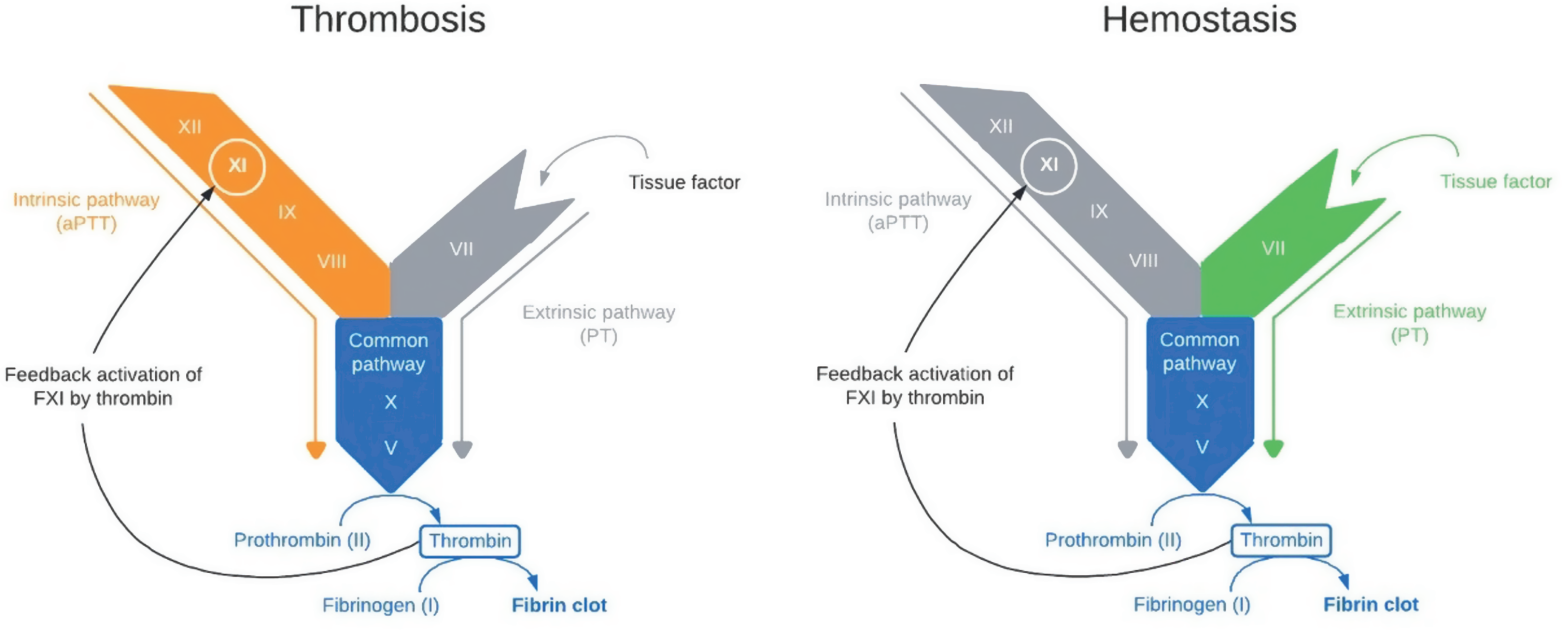
The role of factor XI (FXI)in the coagulation cascade in physiological hemostasis and pathological thrombosis. Thrombosis: Thrombus growth depends on activation of FXI to amplify the process (the color-highlighted pathways are the predominant pathways here, including intrinsic and common pathways, while the non-dominant pathways are grayed-out). Thrombin and FXIIa are important activator of FXI. Hemostasis: FVII or FVIIa binds to extravascular TF leading to explosive thrombin generation to convert fibrinogen to fibrin (the color-highlighted pathways are extrinsic and common pathways and are the predominant pathways here, while the non-dominant pathways are grayed-out). While FXI is also activated by thrombin generated early in hemostasis and contributes to sustained thrombin generation, the intrinsic pathway is not predominantly needed, explaining why FXI is not as crucial in hemostasis.

Factor XI (FXI) inhibitors have the potential to be safer than DOACs. FXI is a key component of the intrinsic coagulation pathway (Fig. 2). Intrinsic pathway plays an important role in cancer-associated hypercoagulability, as shown by a recent study that extracellular vesicles from various cancer cell lines can activate FXII and initiate the intrinsic pathway.^36^ FXI can be activated by FXIIa or by thrombin. Thrombosis is triggered by tissue factor, which induces the generation of small amounts of thrombin. Feedback activation of FXI amplifies thrombin generation, which drives thrombus expansion and stabilization. Therefore, FXI is thought to be essential for thrombosis.

In contrast to its essential role in thrombosis, FXI is likely dispensable for hemostasis, which is a primarily extravascular event whereby the hemostatic plug forms at sites of vessel wall injury to seal the leak. Hemostasis is triggered by the high concentrations of tissue factor in the adventitia, the hemostatic envelope that surrounds blood vessels. These high concentrations of tissue factor trigger explosive thrombin generation much like the rapid clotting observed when tissue thromboplastin is added to plasma to measure the prothrombin time. With such explosive thrombin generation, there is little need for amplification by thrombin-mediated activation of FXI. Therefore, FXI inhibitors have the potential to attenuate thrombosis with little or no disruption of hemostasis (Fig. 2).

Epidemiological data support the role of FXI in thrombosis. Elevated FXI levels were associated with an increased risk of VTE compared with lower FXI levels (odds ratio [OR] 2.2, 95% CI, 1.5-3.2) in a large population-based ­case-control study.^37^ Likewise, using data from 371 695 participants in the United Kingdom Biobank and 2 large-scale genome-wide association studies, genetic disposition to lower FXI levels was associated with reduced risks of venous thrombosis (OR 0.1, 95% CI 0.07-0.14; *P* = 3 × 10^−43^) and ischemic stroke (OR 0.47, 95% CI 0.36-0.61; *P* = 2 × 10^−8^) without an increased risk of major bleeding (OR 0.7, 95% CI 0.45-1.04; *P* = .0739).^38^ These findings are in keeping with the observation that, the risk of ischemic stroke and VTE is lower in patients with congenital FXI deficiency than in the general population. The findings also align with the fact that patients with congenital FXI deficiency have only a mild bleeding diathesis; spontaneous bleeding is rare, although bleeding can occur with surgery or trauma.^39-41^ Therefore, FXI is an attractive target for new anticoagulants.

Four FXI inhibitors have been investigated in phase II RCTs for VTE prevention after knee replacement: An antisense oligonucleotide (fesomersen), 2 monoclonal antibodies (osocimab, which inhibits FXIa, and abelacimab, which binds FXI and locks it in its inactive precursor form such that it cannot be activated by FXIIa or thrombin), and one small molecule FXIa inhibitors (milvexian) (Table 2).^42-45^ Fesomersen, which blocks hepatic synthesis of FXI, requires 3-4 weeks of subcutaneous administration to lower FXI levels into the therapeutic range effect. In contrast, milvexian has a rapid onset of action after oral administration as do the FXI-directed antibodies if given intravenously. All trials compared the FXI inhibitors with enoxaparin (apixaban was used as an additional exploratory control in the osocimab trial). Non-inferior or superior antithrombotic efficacy with selected doses has been shown with all FXI inhibitors (Table 3). The risk of clinically relevant bleeding was low with effective doses of the FXI inhibitors (1%-2%) and similar to that with enoxaparin. The observation that FXI inhibitors were at least as effective as enoxaparin, which inhibits FXa and thrombin, suggests that even though VTE is likely triggered by tissue factor exposed at the surgical site, feedback activation of FXI by thrombin appears to be an important driver, a process that is blocked with FXI inhibition.

**Table 2.** Randomized controlled trials of factor XI inhibitors in elective knee arthroplasty (trial characteristics).

Characteristics	Buller et al^42^	Weitz et al^43^	Verhamme et al^44^	Weitz et al^45^
FXI inhibition	Antisense oligonucleotide (FXI-ASO)	Monoclonal antibody (Osocimab)	Monoclonal antibody (Abelacimab)	Macrocyclic small molecule inhibitor (Milvexian)
Mechanism of action	Reduces FXI synthesis, thereby lowering FXI levels	Binds adjacent to the active site of FXIa and inhibits FXIa	Binds FXI and prevents FXI activation by FXIIa or thrombin	Binds to the active site of FXIa
Administration	Subcutaneous	Intravenous	Intravenous	Oral
Number of patients	300	813	412	1242
Doses	200 mg	0.3 mg/kg	30 mg	25 mg twice daily
	300 mg	0.6 mg/kg	75 mg	50 mg twice daily
	Total of 9 doses, first dose (day 1) 36 days pre-surgery, then given days 3, 5, 8, 15, 22, 19, 36, and 39	1.2 mg/kg	150 mg	100 mg twice daily
		1.8 mg/kg	One dose 4-8 hours after surgery	200 mg twice daily
		One dose either before or after surgery		25 mg once daily
				50 mg once daily
				200 mg once daily
				For 10-14 days post op
Comparison	Enoxaparin 40 mg for ≥8 days post op	Enoxaparin 40 mg or apixaban 2.5 mg twice daily for ≥10 days post op or until venography	Enoxaparin 40 mg for 8-12 days post op (until venography)	Enoxaparin 40 mg for 10-14 days post op
Primary efficacy	VTE by bilateral venography 8-12 days after surgery or symptomatic	VTE by bilateral venography 10-13 days after surgery or symptomatic	VTE by unilateral venography 8-12 days after surgery or symptomatic	VTE by unilateral venography 10-14 days after surgery or symptomatic
Principal safety	Major bleeding and CRNMB to day 136	Major bleeding and CRNMB to days 10-13	Major bleeding and CRNMB to day 30	Bleeding of any severity during treatment period plus 2 days

Detailed criteria for major bleeding at surgical site: Bleeding leading to intervention, hemodynamic instability, delayed mobilization or wound healing, prolonged hospitalization, deep wound infection, or hemarthrosis. Major bleeding by ISTH criteria and significant bleeding at surgical site.

Abbreviations: CRNMB, clinically relevant non-major bleeding; ISTH, International Society on Thrombosis and Haemostasis; VTE, venous thromboembolism.

**Table 3.** Randomized controlled trials of factor XI inhibitors in elective knee arthroplasty (outcomes).

Outcomes	Buller et al^42^	Weitz et al^43^	Verhamme et al^44^	Weitz et al^45^
VTE				
Factor XI inhibitor	200 mg: 27% 300 mg: 4%	Post op-	30 mg: 13%	25 bid: 21%
		0.3 mg/kg: 23.7%	75 mg: 5%	50 bid: 11%
		0.6 mg/kg: 15.7%	150 mg: 4%	100 bid: 9%
		1.2 mg/kg: 16.5%		200 bid: 8%
		1.8 mg/kg: 17.9%		25 qd: 25%
		Pre-op-		50 qd: 24%
		0.3 mg/kg: 29.9%		200 qd: 7%
		1.8 mg/kg: 11.3%		
Enoxaparin	30%	Enox: 26.3% Apix: 14.5%	22%	21%
Interpretation (compared with Enox)	200 mg was non-inferior 300 mg was superior	All doses other than 0.3 mg/kg were non-inferior Pre-op 1.8 mg/kg dose was superior	30 mg was non-inferior 60 and 75 mg were superior	100 bid, 200 bid, 200 qd were superior
Primary safety^*^				
Factor XI inhibitor	200 mg: 3% 300 mg: 4%	Postop-	30 mg: 2%	25 bid: 1%
		0.3 mg/kg: 2%	75 mg: 3%	50 bid: 5%
		0.6 mg/kg: 0%	150 mg: 0%	100 bid: 5%
		1.2 mg/kg: 1%		200 bid: 3%
		1.8 mg/kg: 3%		25 qd: 0%
		Pre-op-		50 qd: 5%
		0.3 mg/kg: 1.9%		200 qd: 6%
		1.8 mg/kg: 4.7%		
Enoxaparin	8%	Enox: 5.9%	0%	4%
		Apix: 2%		
Major bleeding				
Factor XI inhibitor	200 mg: 0% 300 mg: 1%	Post op-	30 mg: 0%	0%
		All doses: 0%	75 mg: 1%	
		Pre-op-	150 mg: 0%	
		0.3 mg/kg: 0%		
		1.8 mg/kg: 0.9%		
Enoxaparin	0%	0% for Enox and Apix	0%	<1%
CRNMB				
Factor XI inhibitor	200 mg: 3% 300 mg: 1%	Post op-	30 mg: 2%	All 1% except for 25 mg (bid or qd) 0%
		0.3 mg/kg: 2%	75 mg: 2%	
		0.6 mg/kg: 0%	150 mg:0%	
		1.2 mg/kg: 1%		
		1.8 mg/kg: 3%		
		Pre-op-		
		0.3 mg/kg: 1.9%		
		1.8 mg/kg: 3.7%		
Enoxaparin	8%	Enox: 5.9%	0%	1%
		Apix: 2%		
Interpretation (compared to Enox)	No differences	No differences	No differences	No differences

^*^The primary safety endpoint is composite of major bleeding and CRNMB in all trials, except for all bleeding (including minor bleeding) by Weitz et al.^43^

Abbreviations: Apix, apixaban; bid, twice daily; Enox, enoxaparin; CRNMB, clinically relevant non-major bleeding; MB, major bleeding; qd, once daily; VTE, venous thromboembolism.

There are more ongoing or upcoming clinical trials to investigate various FXI inhibitors for different indications, which are summarized in Table 4.

**Table 4.** Selected ongoing and upcoming trials of factor XI inhibitors (not including published studies, which were discussed in the text and Tables 2 and 3).

Drug	Mechanism	Study (NCT number)	Route	Indication	*N*	Comparator
IONIS-FXI_Rx_	Antisense oligonucleotide of FXI	EMERALD NCT03358030	SC	ESRD on HD	213	Placebo
Fesomersen (IONIS-FXI-L_Rx_)	Ligand-conjugated (LICA) antisense oligonucleotide	RE-THINc ESRD NCT04534114	SC	ESRD on HD	307	Placebo
Osocimab	Monoclonal antibody against FXIa	CONVERT NCT04523220	SC	ESRD on HD	686	Placebo
		NCT03787368	IV	ESRD on HD	55	Placebo
Abelacimab	Monoclonal antibody against FXI	AZALEA-TIMI 71 NCT04755283	SC	Atrial fibrillation	1200	Rivaroxaban
		ASTER NCT05171049	IV followed by SC	CAT	1655	Apixaban
		MAGNOLIA NCT05171075	IV followed by SC	CAT, GI/GU	1020	Dalteparin
Milvexian	Small molecule inhibitor of FXIa	AXIOMATIC-SSP NCT03766581	Oral	Acute ischemic stroke, TIA (on ASA and clopidogrel^*^)	2366	Placebo
Asundexian	Small molecule inhibitor of FXIa	PACIFIC-AMI NCT04304534	Oral	Acute myocardial infraction on ASA +/−clopidogrel)	1592	Placebo
		PACIFIC- STROKE NCT04304508	Oral	Acute non-cardioembolic ischemic stroke (on antiplatelet therapies)	1808	Placebo
Xisomab 3G3	Monoclonal antibody that blacks FXI activation by FXIIa but not by thrombin	NCT04465760	Intravenous	Prophylaxis for catheter related thrombosis in cancer	50	None (single arm)

^*^ASA and clopidogrel for 21 days followed by ASA alone thereafter.

Abbreviation: ASA, aspirin; AVG, arteriovenous graft; CAT, cancer-associated thrombosis; ESRD, end stage renal disease; GI, gastrointestinal; GU, genitourinary; HD, hemodialysis; IV, intravenous; SC, subcutaneous; TIA, transient ischemic attack.

## Pharmacology of Abelacimab

Abelacimab is a fully humanized monoclonal antibody that binds to FXI with high affinity and locks it in the zymogen confirmation, preventing its activation by FXIIa and thrombin.^46,47^ It has been evaluated in 3 phase I human studies and 2 phase II studies and is found to be safe and promising.^46^ The pharmacokinetics and pharmacodynamic models obtained from phase I/II studies showed that abelacimab has a half-life of approximately 20 days. Intravenous administration produces rapid and dose-dependent inhibition of FXI. No infusions were stopped due to hypersensitivity reactions and no anti-drug antibodies were detected.^46^

## Abelacimab for Treatment of Cancer-Associated VTE

Abelacimab could potentially address most of the unmet needs in the current management of cancer-associated VTE (Fig. 1). The benefit of low bleeding potential of abelacimab, if confirmed, will provide a safer anticoagulant option for patients with high risk of bleeding, as well as those with cancer or in need of concomitant antiplatelet therapy.^46^ The 20-day half-life of abelacimab allows for once monthly subcutaneous dosing, which could improve persistence.^16^ Parenteral administration circumvents the potential for poor drug absorption in cancer patients with GI disturbances. Abelacimab is not cleared via the kidneys or metabolized in the liver.^46^ Therefore, its use is unaffected by severe kidney or hepatic dysfunction. Finally, because catheter-related thrombosis is triggered by activation of FXII, FXI inhibitors may prove more effective than DOACs for prevention or treatment of central venous catheter thrombosis.

Two phase III multicenter RCTs are underway to evaluate the efficacy and safety of abelacimab for the treatment of cancer-associated VTE, the ASTER, and MAGNOLIA trials (Table 4). The ASTER trial (NCT05171049) is comparing abelacimab with apixaban for treatment of VTE in patients with cancer-associated VTE for which a DOAC treatment is intended, whereas the MAGNOLIA trial (NCT05171075) is specifically assessing the efficacy and safety of abelacimab comparing with dalteparin for treatment of VTE in patients with intact, unresectable GI or GU cancers. In both trials, abelacimab is administered as an intravenous infusion on day 1 (to achieve rapid FXI inhibition), followed by monthly subcutaneous injection (to maintain FXI inhibition) for a total of 6 months. The primary efficacy outcome for both trials is the time to first VTE recurrence consisting of new proximal deep vein thrombosis, and new or fatal PE or unexpected death for which PE cannot be excluded. Secondary outcomes include time to the first major or CRNM bleed and net clinical benefit, defined as survival without VTE recurrence, major or CRNM bleeds.

Several caveats are worth noting with the introduction of FXI inhibitors in clinical use. FXI inhibition will result in a prolonged activated partial thromboplastin time (aPTT), as FXI is essential in initiating clot formation in vitro as part of the aPTT assay. This might cause concerns about hemostasis, but aPTT and/or FXI levels are poorly associated with true bleeding phenotypes in patients with congenital FXI deficiency (and remains to be seen in those receiving FXI inhibitors). This is because, unlike what happens in vitro, the physiologic hemostasis in vivo is mainly driven by the tissue factor-FVIIa (Fig. 2).^48^ Therefore, the aPTT is likely to be a poor predictor of the risk of bleeding in patients receiving FXI inhibitors, although it could be a good marker for the presence of the drug.^48^ The resultant prolonged aPTT could also complicate the monitoring of alternative anticoagulants such as unfractionated heparin (if used), which can be addressed by monitoring heparin anti-Xa levels (by chromogenic assays). In addition, some FXI inhibitors such as abelacimab have a long half-life (20 days), while studies to date showed that FXI inhibitors are associated with bleeding rates as low as, or lower than standard anticoagulants, inevitable scenarios such as hemorrhage, urgent procedures, or thrombocytopenia will occur, particularly in patients with cancer, and the optimal management strategies in these settings remain to be elucidated. One recent publication has begun the process of developing a proposal to apply similar approaches as managing those with congenital FXI deficiency patients, including the use of antifibrinolytic agents, fibrin glue, and/or low-dose recombinant factor VIIa.^49^ Fresh frozen plasma or FXI concentrates are less likely to be effective given rapid neutralization of these exogenous FXI by excess free abelacimab. Further development and validation of effective and safe protocols are needed.

## Conclusion

In conclusion, thrombosis remains to be a significant source of morbidity and mortality in patients with cancer. Major advances in anticoagulant treatments, such as DOACs, have significantly raised the bar for effective and safe anticoagulant options in recent years, but further improvement in the care of this vulnerable population is required. Factor XI inhibitors may provide a potential novel approach to address current unmet needs, including the risks of bleeding, DDIs, liver or kidney dysfunction, and long-term persistence in the treatment of cancer-associated thrombosis.

## Data Availability

No new data were generated or analyzed in support of this research.
